# Arginase: shedding light on the mechanisms and opportunities in cardiovascular diseases

**DOI:** 10.1038/s41420-022-01200-4

**Published:** 2022-10-08

**Authors:** Zhuozhuo Li, Liwei Wang, Yuanyuan Ren, Yaoyao Huang, Wenxuan Liu, Ziwei Lv, Lu Qian, Yi Yu, Yuyan Xiong

**Affiliations:** 1 Xi’an Key Laboratory of Cardiovascular and Cerebrovascular Diseases, Xi’an No.3 Hospital, Faculty of Life Sciences and Medicine, Northwest University, Xi’an, Shaanxi China; 2 Key Laboratory of Resource Biology and Biotechnology in Western China, Ministry of Education, School of Medicine, Northwest University, Xi’an, Shaanxi China; 3 Department of Endocrinology, Xi’an No.3 Hospital, the Affiliated Hospital of Northwest University, Northwest University, Xi’an, Shaanxi China

**Keywords:** Mechanisms of disease, Cell death

## Abstract

Arginase, a binuclear manganese metalloenzyme in the urea, catalyzes the hydrolysis of L-arginine to urea and L-ornithine. Both isoforms, arginase 1 and arginase 2 perform significant roles in the regulation of cellular functions in cardiovascular system, such as senescence, apoptosis, proliferation, inflammation, and autophagy, via a variety of mechanisms, including regulating L-arginine metabolism and activating multiple signal pathways. Furthermore, abnormal arginase activity contributes to the initiation and progression of a variety of CVDs. Therefore, targeting arginase may be a novel and promising approach for CVDs treatment. In this review, we give a comprehensive overview of the physiological and biological roles of arginase in a variety of CVDs, revealing the underlying mechanisms of arginase mediating vascular and cardiac function, as well as shedding light on the novel and promising therapeutic approaches for CVDs therapy in individuals.

## Facts

- Arginase is associated with L-arginine metabolism, nitric oxide production, and multiple signal pathways dependently or independently of its L-arginine-urea hydrolase activity.
- Arginase regulating various cellular functions and processes (senescence, apoptosis, proliferation, inflammation, and autophagy) plays key roles in the pathogenesis of cardiovascular diseases, such as hypertension, atherosclerosis, stroke, ischemic reperfusion injury and heart failure.
- Arginase-mediated p38MAPK, mTOR, and p53 and other signaling pathways are involved in the impairment of several cellular functions during the progression of cardiovascular diseases (CVDs).
- Targeting arginase provides a huge research prospect for CVDs therapy.

## Open questions

- What are the exact molecular mechanisms that arginase regulates the cell senescence, apoptosis, proliferation, inflammation, and autophagy?
- What are the specific differences between ARG1 and ARG2 in various cells/tissues?
- Is it feasible to develop ARG1 and ARG2-specific inhibitors?
- What are the adverse effects of targeting arginase in CVDs?

## Introduction

Cardiovascular diseases (CVDs) are the leading cause of death in all regions of the world. In 2019, the mortality rate associated with CVDs was nearly 17.9 million worldwide, representing 32% of all global death [1]. It is estimated that in 2030, deaths from CVDs will exceed 23 million [2]. Cardiovascular diseases, a collective term for cardiac and blood vessel diseases, conclude several conditions, such as hypertension [3], atherosclerosis [4], heart failure (HF), etc. Until now, a great deal of work has been performed to investigate the underlying mechanisms of cardiovascular diseases and corresponding therapeutic interventions. However, the mechanisms mediating the development and progression of CVDs remain incompletely understood.

Arginase, an ubiquitous metalloenzyme with L-arginine hydrolase activity, is capable of hydrolyzing L-arginine to form L-ornithine and urea, and regulating the formation of endothelium-derived vasodilator nitric oxide (NO) production by competing the substrate L-arginine with endothelial NO synthase (eNOS) [5]. More specifically, elevation of arginase activity may cause eNOS-uncoupling, that further leads to reduced NO production and increased formation of reactive oxygen species (ROS) [5]. The lack of NO and overproduced ROS play a key role in the progression of endothelial dysfunction [6], the hallmark of CVDs. Furthermore, arginase can regulate cellular functions including senescence, apoptosis, autophagy, proliferation, and inflammation in multiple cell types independently or dependently on its L-arginine hydrolase activity, which further results in the pathophysiological of several CVDs.

In recent years, a vast body of evidence convincingly indicated that arginase is closely related to the development of CVDs in human beings, such as hypertension [7], atherosclerosis [8], diabetic vascular disease [9], ischemic reperfusion (IR) [10], and HF [11]. Furthermore, some small-molecular arginase inhibitors have been explored and clinically tested in hypertension, HF, IR, etc. Therefore, arginase was gradually regarded as a novel and promising therapeutic target for various CVDs. In this review, we summarize the features and functions of arginase, the roles of arginase in cells of vascular and cardiac system, as well as in the development of CVDs. Novel and promising therapeutic approaches that target arginase for CVDs treatment in individuals are also summarized in an effort to preserve cardiovascular health in the background of high global health burden on CVDs.

## Arginase

### Isoforms of arginase

Arginase, an ubiquitous metalloenzyme with L-arginine hydrolase activity, which had been found in bacteria, yeasts, plants, invertebrates, and vertebrates [12], plays a critical role in physiological and pathological conditions [13]. There are two isoforms of arginase: arginase 1 (ARG1), which is expressed in the cytosol, and arginase 2 (ARG2), which is mainly located in the mitochondria. Although there are approximately 60% homologous between the two isoforms, they show 100% homology in the critical areas for catalyzing function [14], and exhibit similar enzymatic activity. However, the two isoforms differ in their tissue distribution and physiological functions [5]. Both arginase isoforms were found in mammals, whereas most plants, bacteria, yeasts, and invertebrates only express ARG2 [13]. ARG1 is mainly expressed in the liver responsible for the urea cycle, and also found in a variety of extrahepatic tissues including cardiovascular system, red blood cells (RBCs), skin, specific immune cell populations, lung, and intestine [5, 15–18], whereas the specific functions of ARG1 in these tissues remain unknown. ARG2, on the other hand, is significantly expressed in the kidney and also widely expressed in some other organs like the brain and retina [13].

### Functions: L-arginine metabolism

**Fig. 1:**
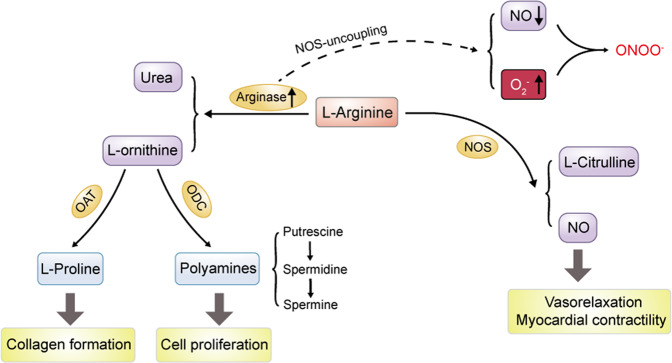
Role of arginase metabolism in regulating polyamine and NO production. Arginase converts L-arginine to urea and L-ornithine that is further metabolized by ODC to polyamines including putrescine, spermidine, and spermine, which play a significant role in cell proliferation from cardiovascular system, or is converted by OAT to L-proline that is essential for collagen formation. Meanwhile, L-arginine also serves as substrate of the enzyme NOS which specifically catalyzes L-arginine to L-citrulline and NO which can promote vasorelaxation and myocardial contractility. The increase of arginase may cause NOS-uncoupling, which contributes to decreased NO production and increased O_2_^−^ and ONOO^−^. ODC ornithine decarboxylase, OAT ornithine aminotransferase, NOS nitric oxide synthase, O_2_^−^ superoxide anion, NO nitric oxide, ONOO^−^ peroxynitrite.

Arginase specifically catalyzes the conversion of the amino acid L-arginine to L-ornithine and urea to dispose of toxic ammonia in the final step of the urea cycle (Fig. 1) [19]. L-ornithine can be further metabolized by ornithine decarboxylase to small cationic molecules polyamines. Polyamines are involved in a variety of fundamental functions including cell proliferation, cell growth, and transport [20], or catalyzed by ornithine aminotransferase to form L-proline, which is essential for collagen formation [13]. Notably, L-arginine also serves as the sole nitrogen-containing endogenous substrate of the enzyme nitric oxide synthase (NOS), which specifically catalyzes the conversion of L-arginine to L-citrulline, nitric oxide, and reactive nitrogen intermediates, such as peroxynitrite [21]. It is well known that NO, an unorthodox messenger molecule, plays essential roles in many biological functions, especially in the circulatory system, such as vasodilation regulation, inhibition of platelet aggregation and adhesion, inhibition of leukocyte adhesion and vascular inflammation, and control of vascular smooth muscle proliferation [22]. Thus, L-arginine is also critical for maintaining endothelial function and preventing CVDs [23]. For example, supplementation of L-arginine reduces IR-induced endothelial dysfunction in patients with type 2 diabetes mellitus (T2DM) and coronary artery disease (CAD) [24]. Yan et al. demonstrated that this supplementation can boost NO bioavailability through inducing higher expression and phosphorylation of eNOS [25]. Moreover, L-arginine has been shown to inhibit inflammatory activation and regulate redox homeostasis in interstitial aortic valve cells to suppress the pro-calcific differentiation [26]. Accordingly, L-arginine supplementation for prevention and treatment of cardiovascular disorders, such as hypertension, atherosclerosis, CAD, HF, and peripheral artery disease, are receiving widespread attention, although its clinical efficacy still remains debatable [23]. And those controversial results could be explained by asymmetric dimethylarginine (ADMA), an endogenous inhibitor of NOS. It was reported that L-arginine level and L-arginine/ADMA ratio are positively related to endothelium-dependent vasodilation in resistance arteries [27], and L-arginine/ADMA ratio is now regarded as a crucial marker of NO bioavailability and risk factor of atherosclerosis [28].

In view of the importance of NO and L-arginine in endothelial function, the imbalance between arginase and NOS due to arginase upregulation can induce the overconsumption of L-arginine, provoking a lack of substrate for NOS to produce NO [19, 29], leading to endothelial dysfunction. Moreover, decreased bioavailability of L-arginine also induces NOS-uncoupling, which produces superoxide anion instead of NO, and the increased oxidative stress further exacerbates NOS-uncoupling [5], and aggravates NO inactivation. Furthermore, ARG2 promotes Ca^2+^-dependent eNOS phosphorylation through parkin-dependent p32 degradation [30]. As a result, an imbalanced increase in arginase in the cardiovascular system, particularly in ECs, will limit NO production and promote superoxide formation, eventually leading to vascular endothelial dysfunction.

### Arginase expression in cardiovascular system

#### Arginase expression in cardiac system

Both ARG1 and ARG2 were reported to express in the cardiac system, including myocytes and nonmyocytes (Table 1). Jung et al. reported that both arginase isozymes were detected in left ventricular (LV) crude homogenates, however, only ARG1 was present in isolated cardiac myocytes, which was downregulated in LV hypertrophy, and ARG2 was found in nonmyocytes [31]. Conversely, in rats, although both ARG1 and ARG2 were present in the whole heart, ARG2 alone was found in isolated myocytes [32], indicating that the cardiac arginase expression appears to be species- and tissue-dependent. Advanced studies in heart found that ARG1 expression can be induced by hypobaric hypoxia [33], and ARG2 was significantly upregulated in the aged cardiac tissue [11]. Arginase in cardiac myocytes is capable of inhibiting the inflammation response [34] and limiting NO as well as cGMP synthesis, hence regulating myocardial contractility [31]. Furthermore, arginase was also detected in the immune cells infiltrating in heart. For instance, both ARG1 and ARG2 were found in the polymorphonuclear cells in ischemic-reperfused myocardium [35], and ARG1 expression was observed in heart-infiltrated CD68^+^ macrophages [36]. Arginase appears to have a role in the control of the immune response in the heart and the development of cardiac disease [36].

**Table 1:** Expression and function of arginase in cardiovascular system.

Type of cell	Arginase expression	Species/tissue/cell	Inducer or activator	Effect of arginase	Refs
Vascular system					
EC	ARG1	Human coronary arterioles	Increased in diabetes	Reduce NO production and diminish vasodilation	[55]
		Mouse coronary arterioles	Increased by TNF-α	Lead to O_2_^−^ production and induce endothelial dysfunction in IR injury	[48]
		HUVEC	—	Promote endothelial senescence and inflammatory responses through eNOS-uncoupling	[45]
		BAEC	Increased by oxidative species through RhoA/Rho kinase pathway	—	[46]
		RAEC	Increased by thrombin	—	[47]
		MAEC	Increased in HFHS diet	Regulate obesity-induced vascular inflammation	[37]
		BREC	Induced by NOX2	Induce premature EC senescence	[43]
	ARG2	HUVEC	—	Inhibit EC autophagy through MTOR and PRKAA/AMPK signaling	[8]
		Rat coronary artery	Increased in diabetes	Impair coronary artery microvascular function	[39]
		HUVEC	Increased by ageing	Activate p38MAPK and S6K1; promote endothelial senescence	[54]
		Porcine carotid endothelial cell	Increased by exposure of arteries to OSS	Impair NO-dependent endothelial function	[53]
		Human carotid artery endothelial cell	Stimulated by ET-1	Stimulate ROS formation in THP-1-derived macrophages	[51]
		HAEC	Activated by OxLDL through LOX-1 receptor and Rho/ROCK signaling	Downregulate NO, increase ROS, lead to EC dysfunction	[52]
		HPMVEC	Increased by hypoxia	Promote HPMVEC proliferation	[41]
VSMC	ARG1	RASMC	—	Promote RASMC proliferation through polyamines production	[84]
		Human aortic	—	Suppresses TNF-α release, inhibit monocyte chemotaxis and migration, inhibit NO release from iNOS, attenuate atherosclerotic plaque inflammation	[181]
	ARG2	HPASMC	Inhibited by cAMP	Promote HPASMCs proliferation	[42]
		HUVSMC	—	Promote VSMC senescence and apoptosis by activating mTORC1 through myo1b	[57]
		HPASMC	Increased by IL-13	Induce HPASMCs proliferation and pulmonary vascular remodeling	[61]
		HPASMC	Increased by AMPK	Promote HPASMCs proliferation	[87]
		HUVSMC	—	Promote VSMC senescence and apoptosis through p66shc and p53	[69]
Macrophage	ARG1	—	Inhibited by Fra-1	Inhibit inflammation in arthritis through fostering the transition of macrophages from a pro-inflammatory to an anti-inflammatory state	[93]
		—	Induced by lipoproteins	—	[92]
	ARG2	—	Induced by ET-1	Promote ROS production	[51]
		—	—	Promote macrophage pro-inflammatory responses through mitochondrial reactive oxygen, result in atherogenesis	[94]
		—	Increased by IL-10	Enhance SDH activity, increase mitochondrial respiration, influence the inflammatory status of the macrophage	[182]
Cardiac system					
Myocardial	ARG1	Cat	Decreased in LVH	Regulate NO-dependent myocardial contractility	[31]
	ARG2	Rat	Increased in ageing	Decrease age-related contractile function	[11]
		Rat	—	Regulate NO-dependent basal myocardial contractility in a NOS1-dependent manner.	[32]
Polymorphonuclear	ARG1; ARG2	Pig	Increased in IR	—	[35]

*ARG1* arginase 1, *ARG2* arginase 2, *VSMC* vascular smooth muscle cell, *EC* endothelial cell, *NO* nitric oxide, *TNF-α* tumor necrosis factor-α, *IR* ischemic reperfusion, *eNOS* endothelial NO synthase, *HFHS* high-fat/high-sucrose, *NOX2* NADPH oxidase 2, *p53* tumor suppressor p53, *MTOR* mammalian target of rapamycin, *PRKAA/AMPK* protein kinase AMP-activated α catalytic subunit, *p38MAPK* p38 mitogen-activated protein kinase, *S6K1* ribosomal protein S6 kinase, *OSS* oscillatory shear stress, *ET-1* Endothelin-1, *OxLDL* oxidized low-density lipoprotein, *ROS* reactive oxygen species, *iNOS* inducible nitric oxide synthase, *mTORC1* mechanistic target of rapamycin complex 1, *IL-13* interleukin-13, *Fra-1* Fos-related antigen 1, *IL-10* interleukin-10, *SDH* succinate dehydrogenase, *LVH* left ventricular hypertrophy, *HUVEC* human umbilical vein endothelial cell, *BAEC* bovine aortic endothelial cells, *RAEC* rat aortic endothelial cells, *MAEC* mouse aortic endothelial cell, *HAEC* human aortic endothelial cells, *HPMVEC* human pulmonary microvascular endothelial cells, *HPASMC* human pulmonary artery smooth muscle cell, *HUVSMC* human umbilical vein smooth muscle cell.

#### Arginase expression in vascular system

Numerous studies proved that arginase is widely expressed in a variety of blood vessels, including the aorta [37], carotid [38], coronaries [39], gracilis muscle arterioles [40], pulmonary microvascular [41], pulmonary artery [42], and retinal microvascular (Table 1) [43]. Both isoforms of arginase can be detected in various ECs of different species, such as human pulmonary artery ECs [44], human umbilical vascular endothelial cells (HUVECs) [8, 45], bovine aortic ECs [46], rat and mouse aortic ECs [37, 47], coronary arteriolar ECs [39, 48] and so on. In ECs, expression of ARG1 can be stimulated by thrombin [47], oxidative species, NADPH oxidase 2 [49], and tumor necrosis factor-alpha (TNF-α) [48], while ARG2 can be regulated by hypoxia [50], ET-1 [51], oxidized low-density lipoprotein [52], oscillatory shear stress [53], and aging [54]. Both ARG1 and ARG2 in ECs have been demonstrated to be involved in the regulation of NO production and vasodilation [55, 56]. Arginase was also found to be present in VSMCs [57]. ARG1 from VSMCs can be activated by interleukin-4, interleukin-13 [58], transforming growth factor-β1 [59], and cyclic stretch [60]. The pro-inflammatory factors interleukin-13 [61], and hypoxia [42] can induce the expression of ARG2 in VSMCs.

## Arginase mediates various cellular functions

### Arginase in cellular senescence

**Fig. 2:**
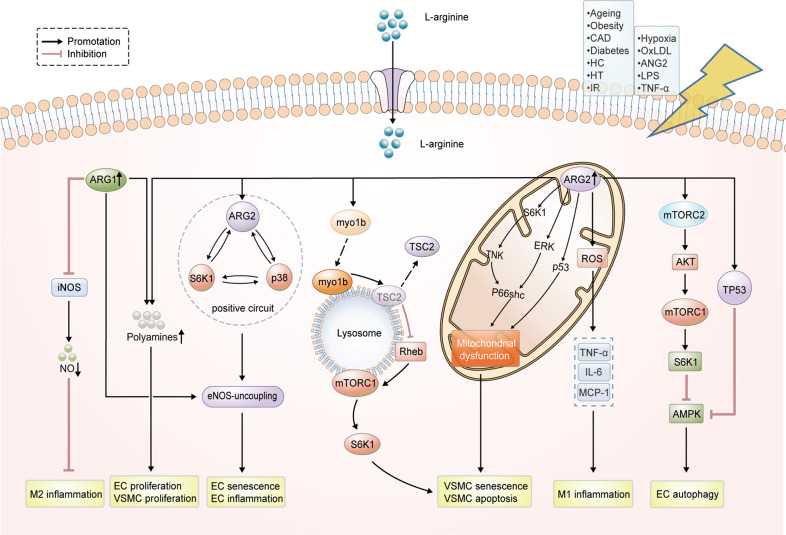
Arginase regulates cellular functions in cardiovascular system through L-arginine metabolism and multiplicate signaling pathways. Arginase activity and expression can be stimulated by hypoxia, OxLDL, ANG2, LPS, TNF-α, also upregulated in ageing, obesity, CAD, diabetes, HC, HT, and IR. Both ARG1 and ARG2 can promote EC/VSMC proliferation through polyamine synthesis. ARG1 promotes M2 anti-inflammation response through blocking NO production from iNOS, but ARG2 induces pro-inflammatory cytokine production (TNF-α, IL-6, and MCP-1) in M1 via mitochondrial ROS production. ARG1 can induce EC senescence and inflammation through eNOS-uncoupling. The positive crosstalk of ARG2, p38MAPK, and S6K1 can accelerate vascular endothelial senescence through eNOS-uncoupling. ARG2 promotes myo1b association with lysosomes, induces lysosome re-distribution and TSC-lysosome dissociation, reliefs the inhibitory effect of TSC2 on the mTOR activator Rheb therefore actives mTORC1-S6K1 signaling, eventually leading to VSMC senescence. ARG2 promotes mitochondrial dysfunction by complex positive crosstalk among S6K1‐JNK, ERK, p66Shc, and p53, leading to VSMC senescence and apoptosis. ARG2 can activate mTORC2, further activating AKT-mTORC1-S6K1 signaling and inhibiting AMPK, thereby impairing cellular autophagy. Besides, ARG2 can inhibit AMPK activation through TP53 signaling. ANG II angiotensin II, LPS lipopolysaccharide, OxLDL oxidized low-density lipoprotein, TNF-α tumor necrosis factor-alpha, CAD coronary artery disease, HC hypercholesterolemia, HT hypertension, IR ischemic reperfusion, VSMC vascular smooth muscle cell, EC endothelial cell, ARG1 arginase 1, ARG2 arginase 2, IL-6 interleukin-6, MCP-1 monocyte chemoattractant protein-1, ROS reactive oxygen species, p53 tumor suppressor p53, mTOR mammalian target of rapamycin, p38MAPK p38 mitogen-activated protein kinase, mTORC1 mechanistic target of rapamycin complex 1, myo1b myosin-1b, TSC tuberous sclerosis complex, ERK extracellular signal‐regulated kinase, JNK c‐Jun N‐terminal kinase, TP53 tumor protein 53, AKT v-akt murine thymoma viral oncogene homolog 1, AMPK protein kinase AMP-activated α catalytic subunit, S6K1 ribosomal protein S6 kinase.

Cellular senescence, a state of permanent and irreversible cell-cycle arrest [62], is intimately linked to CVDs and was found in sick cardiovascular tissues [63]. Excessive arginase activity has been associated with EC senescence in aging humans and animals [64], and lowering arginase protein expression/activity reverses endothelial senescence [65, 66]. Overexpression of ARG1 can promote cell senescence indicated by upregulation of senescence markers p53-S15 and p21^Cip1^, and increased SA-β-gal positive cells in HUVECs (Fig. 2) [45]. Diabetes-induced premature senescence of retinal ECs was alleviated by inhibition of ARG1 [43]. Similarly, ARG2 was also found to be upregulated in senescent ECs compared to young cells, and its silencing suppressed the senescence markers such as SA-β-gal activity, p53-S15, and p21 [67]. Chronic L-arginine treatment accelerates EC senescence, which is also through upregulation of ARG2 [68]. The mechanism of arginase regulating endothelial senescence is believed to be related with the increase oxidative stress because of eNOS-uncoupling [45, 54, 67]. Further studies discovered that both ARG1 and ARG2 activated the mammalian target of rapamycin complex 1 (mTORC1)/S6 protein kinase 1 (S6K1) pathway which is an important regulator of organism aging. Moreover, ARG2 was also identified to form a positive circuit together with S6K1 and p38 mitogen-activated protein kinase (p38MAPK), contributing to endothelial senescence and cardiovascular aging through eNOS-uncoupling [54]. In VSMCs, arginase also exhibited pro-senescence function. Xiong et al. have reported that overexpression of ARG2 in VSMCs from human umbilical veins can induce mitochondrial dysfunction, and finally lead to the senescence through a mechanism involving activation of p66Shc and p53 [69]. Additionally, ARG2 accelerates VSMC senescence through mTORC1-S6K1 activation, which is mediated by association of myo1b with lysosomes and lysosome re-distribution [57]. Of note, these functions of ARG2 including activation of S6K1, p38MAPK, and p66shc appear to be independent of its L-arginine-urea hydrolase activity, but rely on its C-terminal [57, 69]. Besides in the circular system, arginase was also widely found to involve in cell senescence in other systems. For example, overexpression of ARG2 in keratinocytes can enhance the expression of senescence-associated β-Gal [70]. ARG2 exacerbates nucleus pulposus cell senescence caused by oxidative stress and inflammation via the nuclear factor-κB pathway [71]. Importantly, disruption of ARG2 gene was shown to lengthen the longevity of mice [72]. Thus, the arginase may present a promising target to reverse vascular cell senescence, slow down the aging process and mitigate the development of CVDs. However, the specific mechanism of arginase regulating senescence still needs further study.

### Arginase in cell apoptosis

Apoptosis is a type of programmed cell death that is distinct from necrosis [73]. Excessive cellular apoptosis in cardiovascular system may contribute to a fatal outcome [74]. Arginase has been reported to regulate apoptosis in various cells. In VSMCs, ARG2 can induce apoptosis due to mitochondrial dysfunction through a complex positive crosstalk among S6K1-c-Jun N-terminal kinase, extracellular-signal-regulated kinase, p66Shc, and p53, independently of its ureohydrolase activity (Fig. 2) [69]. Suwanpradid et al. demonstrated that ARG2 is associated with apoptosis of bovine retinal endothelial cells and vascular injury induced by hyperoxia, and inhibition of ARG2 activity can minimize apoptosis and ameliorate this scenario by preventing NOS uncoupling [75]. In the old wild-type mice, upregulated ARG2 is engaged in β-cell apoptosis by inducing the release of TNF-α from pancreatic acinar cells through p38MAPK [76]. In addition, arginase can also induce apoptosis through L-arginine deprivation and ROS production in some argininosuccinate synthase defective cells that cannot synthesize L-arginine by themselves [77, 78]. However, there are still some contrary reports about the role of arginase in apoptosis. For example, ARG2 was reported to negatively regulate mercury-induced apoptosis in renal cells [79]. Moreover, upregulated ARG2 protein induced by inflammation can temper cytokine-induced apoptosis in intestinal epithelial cells through reducing NO production competitively, considering that excess NO production can lead to apoptosis [80]. Thus, the regulation of arginase on apoptosis likely depends on the cell type and its mechanism, and the function and mechanism of arginase in cardiovascular apoptosis still need further exploration.

### Arginase in cell proliferation

Cell proliferation is essential for the development of the organism and its organs [81]. Cardiovascular cell proliferation is critical for cardiovascular function and development [82]. As explained above, arginase is capable of converting L-arginine to L-ornithine which is further to form polyamines. Polyamines are necessary for cell proliferation. Complement factor B-induced upregulation of ARG1 can drive polyamine overproduction in macrophages, thereby leading to cardiac fibroblast (CF) proliferation and collagen production [83]. Elevated ARG1 has been shown to boost polyamine production in rat aortic smooth muscle cells, which speeds up VSMC proliferation and neointima formation (Fig. 2) [84]. Silencing ARG2 in senescent VSMCs can diminish proliferation, indicating that ARG2 may promote VSMC proliferation [69]. Decrease of ARG1 and ARG2 activity induced by limonin can prevent native low-density lipoprotein-induced VSMC proliferation in a p21Waf1/Cip1-dependent manner through inactivation of NADPH oxidase [85]. In addition, overexpression of either ARG1 or ARG2 can facilitate endothelial proliferation through polyamine synthesis, which is tightly linked to angiogenesis and wound healing [86]. Hypoxia has been reported to accelerate human pulmonary microvascular ECs proliferation via upregulation of ARG2 by epidermal growth factor–epidermal growth factor receptor–extracellular-signal-regulated kinase pathway [41] and induce human pulmonary artery SMCs proliferation through arginase induction by AMPKα_1_ signaling [87]. On the other side, arginase’s enzymatic activity results in the consumption of L-arginine which is required in some types of cells, resulting in delayed growth and even death. Hepatic ischemia-reperfusion leads to substantial release of arginase and then deprives systemic L-arginine, which further inhibits the proliferation of brain microvascular endothelial cells by cell cycle arrest [88]. Overall, arginase is engaged in the regulation of cell proliferation and has significant implications for angiogenesis, vascular remodeling as well as cardiovascular functions.

### Arginase in cellular inflammation

Inflammation is a natural defense mechanism of the body in response to harmful stimuli [89]. Chronic inflammation, is related to various CVDs, such as atherosclerosis, chronic HF, and myocardial infarction [90]. Due to inhibition of NO from iNOS by arginase, ARG1 is always considered as a classical anti-inflammatory marker in M2 macrophage and is capable of dampening the macrophage inflammatory response. It is reported that recombinant human ARG1 can inhibit the immune function in activated macrophages [91]. Moreover, the expression and activity of ARG1 in macrophages can be boosted by lipoproteins [92] and decreased by Fos-related antigen 1 [93]. Unlike the anti-inflammatory effects of ARG1, ARG2 appears to be more pro-inflammatory. Although ARG2 competes with iNOS to reduce NO, it is nonetheless linked to the M1 phenotype of macrophages. Ming et al. showed that overexpression of ARG2 in macrophages boosts pro-inflammatory cytokine production, such as TNF-α, interleukin-6 (IL-6), and monocyte chemoattractant protein-1 expression by stimulating mitochondrial ROS generation (Fig. 2) [94]. Liu et al. also discovered that the release of TNF-α and IL-6 from bone marrow-derived macrophages of ARG2 knockout mice is lower than that of wild type [95]. Moreover, ARG2 also induces a pro-inflammatory phenotype in other cell types. For example, ARG2 is mediated in senescent ECs to induce expression of inflammatory vascular adhesion molecule-1 (VCAM-1) and intercellular adhesion molecule-1 (ICAM-1) [67], as well as the generation of pro-inflammatory cytokines such as IL-6 and interleukin-8 [54], which is through activation of p38MAPK and S6K1. Huang and colleagues reported that ARG2 promotes age-associated adipose tissue inflammation by inducing IL-6 production through p38MAPK [96]. In pancreatic acinar cells of aging mice, the upregulated ARG2 induces the paracrine release of inflammatory cytokine TNF-α through activating p38MAPK signaling [76]. The level of ARG2 expression in mice is upregulated in proximal tubular S3 segment cells with aging, then silencing ARG2 gene can decrease TNF-α-induced ICAM-1 and VCAM-1 level in the proximal epithelial cells [97]. Downregulating arginase levels can attenuate the increase of inflammatory cytokines, such as TNF-α, IL-6, and interleukin-1β, which mitigates pneumoperitoneum-induced inflammation [98]. Taken together, these data indicate that arginase plays a crucial role in cellular inflammation, but different isoforms of arginase may show different effects, which is most likely related to the activity-independent function of ARG2. Consequently, it will be an interesting challenge to further explore the mechanism of arginase regulating inflammation and its activity-independency effect, which will help to unravel the mystery.

### Arginase in cellular autophagy

Autophagy, a major intracellular degradation system, is implicated in maintaining the healthy status of an organism under stress conditions [99]. A great deal of work has been performed to support a crucial role of autophagy in the regulation of intracellular homeostasis in most cells of cardiovascular origin [100]. In the heart, autophagy primarily serves as a pro-survival process during cellular stress, eliminating aggregated protein and damaged organelles. EC-intrinsic autophagy regulates EC response to a variety of metabolic stresses and is important for redox homeostasis and EC plasticity, and is also involved in modulating other important functions, such as NO production, angiogenesis, and hemostasis/thrombosis [101]. Most recently, arginase has been discovered to play a role in autophagy control. In human ECs, ARG2 impairs cellular autophagy through activation of mTORC2, which further induces AKT-mTORC1-S6K1 signaling and inhibits AMPK, independently of its L-arginine-urea hydrolase activity (Fig. 2) [8]. Moreover, it was observed that recombinant human ARG1 downregulated autophagy in activated macrophages, and activation of autophagy alleviated recombinant human ARG1-induced immunosuppression [91]. In the mouse model of oxygen-induced retinopathy, ARG2 deletion prevents hyperoxia-induced retinal vascular injury, which is associated with activation of autophagy [102]. Additionally, upregulation of ARG2 inhibits autophagy in keratinocytes through stimulating cellular senescence [70]. Overall, maintaining optimal autophagy function by targeting arginase is a viable technique for CVDs treatment. However, the functions and regulation of arginase implicated in vascular autophagy are largely unknown, further investigations are needed to determine the mechanism of ARG1 or ARG2 in autophagy regulation.

## Arginase contributes to CVDs development

### Arginase in hypertension

**Fig. 3:**
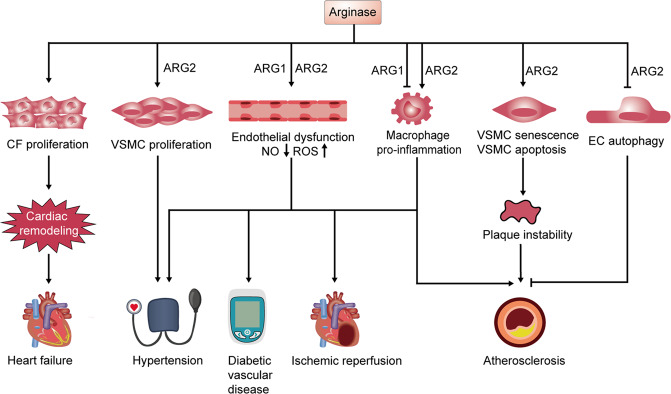
Arginase promotes various CVDs through regulating cellular functions and endothelial dysfunction. Arginase can promote CF proliferation and contribute to cardiac remodeling, which plays a crucial role in heart failure. ARG2 expression can induce the VSMCs proliferation, a hallmark characteristic of hypoxia-induced PAH. Furthermore, upregulated arginase induces endothelial dysfunction via NO reduction and ROS production, which promotes the development of a variety of CVDs such as hypertension, diabetic vascular disease, ischemic reperfusion and atherosclerosis. Moreover, ARG2 leads to atherosclerotic plaque instability through promoting VSMC senescence and apoptosis and dampening endothelial autophagy in atherosclerosis. ARG2 expression triggers macrophage pro-inflammatory responses, resulting in the development of atherosclerosis. CVDs cardiovascular diseases, CF cardiac fibroblast, VSMC vascular smooth muscle cell, EC endothelial cell, PAH pulmonary arterial hypertension, NO nitric oxide, ROS reactive oxygen species.

Hypertension is a well-known risk factor for various CVDs [103]. Arginase-induced endothelial dysfunction has been demonstrated to offer a vital mechanism in the development of hypertension. The age-dependent augment of arginase expression in vascular and plasma occurs before the initiation and progression of hypertension in obese rats, and treatment with an arginase inhibitor prevents the development of hypertension [104]. Subsequently, it was proved that arginase impairs vascular function in elderly spontaneously hypertensive rats via an NO-dependent mechanism (Fig. 3) [7]. In deoxycorticosterone acetate-salt hypertension, arginase is also involved in its pathogenesis, and both arginase isoforms contribute to vascular endothelial dysfunction, especially ARG1 that is upregulated in the aorta of rats with deoxycorticosterone acetate-salt hypertension and associated with the elevated systolic blood pressure (BP), impaired endothelium-dependent vasorelaxation and increased vasoreactivity to constrictor stimuli [105]. Additionally, it was reported that inhibiting arginase improved reflex vasodilatation in patients with hypertension [106], and prevented impaired NO production, and reduced ROS generation in aorta isolated from animals with metabolic syndrome [107]. A recent study reported that ARG2 upregulation activated by hypoxia contributes to a lower NO availability in hypoxia-induced hypertension [108]. In the hypoxia-induced PAH rat model, ARG1 and ARG2 expression were induced [109], and arginase inhibition can mitigate the increase of right ventricle systolic pressure and right ventricle hypertrophy via inhibiting HPASMC proliferation [110]. Similarly, arginase inhibition also decreases the right ventricle systolic pressure and mitigates the lung tissue remodeling in the monocrotaline-induced pulmonary hypertension rat model [111]. Taken together, targeting arginase may be a potential strategy for hypertension therapy and prevention. However, whether ARG1 and ARG2 differ in the development of hypertension warrants further investigation.

### Arginase in atherosclerosis

Atherosclerosis is a common ailment that occurs when plaque builds up inside the arteries. Disease linked to atherosclerosis is the leading cause of death in the world. Endothelial dysfunction as a result of reduced NO bioavailability is a critical initial event in atherosclerosis. Arginase is frequently associated with endothelial dysfunction and further atherosclerosis. For example, Ryoo et al. reported that arginase activity is upregulated in vascular ECs of ApoE^−/−^ mice after a high cholesterol diet, and further investigation revealed that ARG2 knockdown restores decreased vascular NO production, ameliorates endothelial function, and prevents increased vascular stiffness [112]. Importantly, activation of HDAC2 or PARP-1 inhibition is reported to attenuate atherogenesis both through reducing ARG2 expression, restoring NO production in ECs, and ultimately ameliorating vascular dysfunction [113, 114]. Independent of the NO mechanism, Xiong et al. found that ARG2 knockdown suppresses atherosclerotic lesion formation in ApoE^−/−^ mice through rescuing autophagy [8]. Additionally, ARG2 was also demonstrated to affect atherosclerotic plaque instability and progression by promoting VSMC senescence and apoptosis [69] and inducing pro-inflammatory responses in macrophages (Fig. 3) [94]. In addition, in a late stage of human carotid plaques, ARG2 activation in ECs and macrophages can trigger ROS formation, which may further enhance atherosclerosis progression [51]. In contrast to ARG2, ARG1 upregulation has been reported to ameliorate atherosclerosis [115], manifested as dampening atherosclerotic plaque inflammation, increasing Th2 cytokine levels, and facilitating VSMC proliferation, ultimately leading to the elevated atherosclerotic plaque stability [116]. However, Yi et al. showed that arginase inhibition is capable of ameliorating endothelial function through enhancing NO production in ApoE^−/−^mice [117]. These results imply that arginase, especially ARG2, plays a critical role in the pathogenesis of atherosclerosis, which could be a promising therapeutic target for atherosclerosis.

### Arginase in heart failure

HF is defined as a problem with the heart’s ability to pump blood to the rest of the body owning to weakness or stiffness of the heart muscle [118]. Arginase expression and activation play a pivotal role in the development and progression of HF. Clinically, patients with HF are characterized by higher ARG1 expression compared to normal patients [118]. Further study indicated that patients with severe HF showed higher ARG1 expression compared with patients with mild HF, and arginase inhibition ameliorated microcirculation of patients with HF in a NO-dependent manner [118]. Moreover, upregulation of ARG1 stimulated by complement factor B can induce cardiac remodeling in the uremic mice model through causing CF proliferation and collagen production (Fig. 3). In contrast, arginase inhibition tempers cardiac remodeling in uremic mice [83]. A recent study also showed increased ARG1 level in inflammatory cells from blood and heart leads to poor L-arginine bioavailability to eNOS, which might be the reason for HF with preserved ejection fraction in obese ZSF1 rats [119]. Heusch et al. observed that in the rabbit model of pacing-induced HF, ARG2 is significantly upregulated in cardiac and further promotes iNOS uncoupling and superoxide anions production, which may lead to contractile dysfunction [120]. In the doxorubicin‐induced cardiomyopathy mice model, arginase inhibition can promote NO release from aortic ECs and macrophages, decrease the afterload for LV, and eventually ameliorate LV systolic function [121]. Collectively, upregulated arginase plays a causal role in the distinct etiologies of HF, thus arginase may be a potential target for HF treatment.

### Arginase in IR injury

Myocardial IR injury contributes to poor cardiovascular outcomes after myocardial ischemia, cardiac surgery, or circulatory arrest. Accumulating evidence has demonstrated a causal role for arginase in IR injury in vitro and in vivo. In vitro investigation indicated that upregulation of ARG2 in bovine retinal ECs induces mitochondrial dysfunction and fragmentation, increases EC stress, and reduces cell survival, ultimately promoting IR-induced vascular injury, while the specific knockdown of ARG2 in EC of IR mice model with injury in the right eye attenuates IR-induced retinal detachment/edema and reduces impairment of the blood-retinal barrier [56]. In the myocardium and aorta of IR rats, arginase expression is significantly enhanced. Further study showed that vagal nerve stimulation can suppress the IR-induced arginase elevation through activating α7 nAChR, reduce the infarct size and mediate the cardio-protective effect [122]. Arginase reduction induced by ROCK inhibition can reduce infarct size in a NOS-dependent manner, further protecting the diabetic heart against IR injury in rats with type 1 diabetes [123]. Administration of RBCs with elevated arginase expression and activity from mice or patients with T2DM can impair post-ischemic cardiac recovery of nondiabetic hearts via provoking eNOS-derived ROS production in RBCs, while this result can be alleviated by inhibition of RBC arginase [124]. Kövamees et al. reported that arginase inhibition alleviates endothelial dysfunction after IR in patients with CAD [125]. Arginase expression is significantly enhanced in the myocardium of IR pig in comparison with the sham group, and further analysis indicated that arginase downregulation is capable of inducing a decrease in infarct size via increasing NO bioavailability [35], implying that arginase plays a crucial role in the development in IR injury.

### Arginase in diabetic vascular disease

Diabetes is frequently associated with numerous vascular pathologies, which may lead to obvious morbidity and mortality in diabetic patients [13]. Several experimental studies have demonstrated that arginase is a key factor driving cardiovascular complications in diabetic patients. Analysis of forearm blood flow in patients with T2DM showed that arginase inhibition can ameliorate endothelium-dependent vasorelaxation, which is maintained after glucose optimization [9]. ARG1 inhibition can ameliorate streptozocin-induced vascular dysfunction through increased NO production [126]. Studies of the retinal arterioles from diabetic pigs have shown that early type 1 diabetes can increase the arginase activity, thereby impairing endothelium-dependent NOS-mediated dilation of retinal arterioles during diabetes, while arginase blockade leads to the opposite results [127]. Chandra et al. demonstrated that diabetes/hyperglycemic states can cause elevated arginase activation in aortic ECs through RhoA/ROCK/MAPK pathway [128]. Furthermore, Zhou et al. revealed that RBC ARG1 mediates endothelial dysfunction in T2DM. ARG1 expression and activity can be upregulated in RBCs from T2DM by a ROS-dependent mechanism, which leads to endothelial dysfunction in healthy arteries following co-incubation of T2DM RBCs and healthy arteries (Fig. 3) [129]. Further study demonstrated that RBCs peroxynitrite impairs endothelial function in T2DM via activating arginase in endothelial cells [130]. Recently, Li et al. illustrated that serum extracellular vesicle-derived ARG1 levels were significantly higher in T2DM patients compared with non-T2DM patients [131]. Elevated ARG1 in serum exosomes of diabetic mice can be delivered to ECs, which subsequently results in diabetic endothelial dysfunction via inhibiting NO synthesis [132]. Therefore, targeting arginase, especially ARG1 is a promising pharmacological tool to improve vascular function in diabetic patients.

### Arginase in COVID-19 and its vascular complications

Currently, coronavirus disease 2019 (COVID-19) pandemic has led to a global healthcare crisis, and it can cause various systemic complications, including vascular and pulmonary diseases. COVID-19 is caused by the severe acute respiratory syndrome coronavirus 2 (SARS-CoV-2), which entries body through binding angiotensin-converting enzyme 2 (ACE2) that is abundantly expressed in human endothelial cells of the arterial and venous vessels. Thus, endothelial cells are considered as the main barrier against SARS-CoV-2 invasion, and endothelial dysfunction is proved to be a fundamental feature of COVID-19 and associated with COVID-19 complications [133]. For this, arginase is likely to be involved in COVID-19 and its vascular complications, since it induces endothelial dysfunction by depleting L-arginine and decreasing NO bioavailability. Derakhshani et al. reported that the expression of ARG1 was significantly increased in the whole blood of COVID-19 patients compared to healthy individuals, which might be a potential marker in the pathogenesis of COVID-19 and its complications [134]. Furthermore, large amount of ARG1^+^G-MDSC cells was found in the lungs of patients who died from COVID-19 complications, which might deplete L-arginine, leading to impaired T cell receptor and endothelial function [135]. Additionally, numerous investigations based on metabolomics analysis identified lower L-arginine levels in plasma of patients with COVID-19, indicating an increase in arginase activity in these patients [136]. Another clinical study also discovered that circulating level of ADMA in COVID-19 patients is significantly higher, impairing the bioavailability of L-arginine [137]. Based on these, inhibition of arginase and/or replenishment of L-arginine is potential in preventing/treating severe COVID-19. A clinical trial discovered that oral L-arginine supplementation to standard therapy significantly shorten hospitalization of patients with severe COVID-19 [138]. Similarly, another group also indicated that combination of L-arginine and vitamin C shows beneficial effects on long-COVID symptoms [139]. Moreover, L-arginine might attenuate SARS-CoV-2 infection through other mechanisms, such as reducing binding of SARS-CoV-2 to ACE2, inhibiting transmembrane protease serine-type 2, and suppressing SARS-CoV-2 replication [133]. Collectively, targeting abnormal arginase expression and loss of L-arginine might be an effective strategy for prevention and treatment of COVID-19 and its mediated vascular impairment.

## Arginase as a target for CVD therapy

### Chemical arginase inhibitors

Some chemical compounds, such as N-hydroxy-L-arginine (NOHA) and its analogs N-hydroxy-nor-arginine (Nor-NOHA), 2(S)-amino-6-(borono)hexanoic acid (ABH), and S-(2-boronoethyl)-L-cysteine (BEC), were identified as arginase inhibitors which are now being attempted for the treatment of a variety of CVDs (Table 2).

**Table 2:** The effects of arginase inhibitors against cardiovascular diseases.

Arginase inhibitor	Diseases	Model	Dosage	Effect of arginase inhibition	Refs
Chemical arginase inhibitors					
NOHA	IR injury	IR rat	0.1 mmol/L	Decrease inflammatory cells migration in IR, prevent inflammatory cells invasion	[48]
Nor‐NOHA	Cardiomyopathy	Doxorubicin‐induced cardiomyopathy mice	100 μmol/L, 12 h	Facilitate LV systolic function, lower tail BP and afterload for LV	[121]
	PAH	Monocrotaline-induced PH rat	100 mg/kg, 15 d	Reduce RVPsys and lung tissue remodeling	[111]
	Diabetic vascular disease	Retinal arterioles isolated from streptozocin-induced diabetic pigs	0.1 mmol/L, 1.5 h	Improve dilation of retinal arterioles isolated from diabetic pigs	[127]
	Diabetic vascular disease	Patients with T2DM	0.1 mg/min, 2 h	Improve microvascular endothelial function	[145]
	CAD	Patients with CAD	0.1 mg/min, 20 mins	Improve flow-mediated dilatation after IR	[125]
	Hypertension	Spontaneously hypertensive rats	40 mg/day, 10 weeks	Reduce systolic BP, improve vascular function, reduce artery remodeling and cardiac fibrosis	[147]
	Atherosclerosis	ApoE^−/−^ mice	10 mg/kg, 5 days/week, 9 weeks	Reduce the lipid deposition, vascular ROS, the number of macrophages and the size of atherosclerotic plaques	[148]
BEC	PAH	HPAH mouse	1 mM, 2 mL/day	Inhibit HPASMC proliferation, attenuate pulmonary vascular remodeling	[110]
	Obesity	Obese Zucker rats	55.6 μg/hour, 6 d	Normalize BP, restore endothelium-mediated vasodilation	[183]
	Atherosclerosis	ApoE^−/−^ mice	10 μmol/L	Restore endothelial function, reduce plaque burden and plaque load	[112]
ABH	Atherosclerosis	ApoE^−/−^ mice	200 μg/d, 2 weeks	Increase vascular NO and decrease vascular stiffness	[112]
	Hypertension	Male Sprague-Dawley rats	400 μg/kg/ day, 20 d	Reduce elevated BP, revert impaired endothelial-dependent relaxation	[155]
Natural arginase inhibitors					
Animo acids	Diabetic vascular disease	Patients with T2DM	2000 mg/day, 1 month	Restore NO production levels	[161]
	stroke	Ischemic rats	Citrulline (50 mg/kg) or ornithine (200 mg/kg)	Reduce the gait scores, infarct volume and brain edema	[162]
	Hypertension associated with diabetes	Streptozotocin-induced diabetic rats	Citrulline (50 mg/kg) or norvaline (50 mg/kg) or ornithine (200 mg/kg),	Reduce the elevation in diastolic BP, increase NO generation, inhibit ROS generation, restore impairment in vasoconstriction response	[184]
	hypertension	Hypertensive (ISIAH strain) rats	Norvaline (30 mg/kg), 7 d	Reduce the BP	[163]
	Hypertension	Metabolic syndrome rats	Citrulline (50 mg/kg), norvaline (50 mg/kg) and ornithine (200 mg/kg)	Reduce ROS, increase NO, restore endothelial-dependent relaxation, reduce the BP	[107]
Polyphenols	Atherosclerosis	ApoE-KO mice	Pomegranate juice (31 mL/day, 10 weeks)	Promote a switch in macrophage phenotype from M1 pro-inflammatory to M2 anti-inflammatory state	[185]
	IR injury	IR rat	EPI (1 mg/kg, 10 d)	Reduce increased nNOS isoform protein levels, maintain eNOS activity	[176]
	Hypertension	Old rat	PIC (30 mg/kg/day, 4 d)	Reduce BP, enhance NO production, recover endothelial dysfunction	[173]

*ABH* 2(S)-amino-6-(borono)hexanoic acid, *BEC* S-(2-boronoethyl)-L-cysteine, *NOHA* N-hydroxy-L-arginine, *Nor-NOHA* N-hydroxy-nor-arginine, *RSV* resveratrol, *PIC* piceatannol, *EPI* (-)-Epicatechin, *IR* ischemic reperfusion, *LV* left ventricle, *RVP*sys right ventricular systolic pressure, *T2DM* type 2 diabetes mellitus, *CAD* coronary artery disease, *BP* blood pressure, *ROS* reactive oxygen species, *HPASMC* human pulmonary artery smooth muscle cell, *NO* nitric oxide, *ApoE-KO* apolipoprotein E deficient, *ApoE*^*−/−*^ apolipoprotein E-null, *nNOS* neuronal NO synthase, *eNOS* endothelial NO synthase, *PH* pulmonary hypertension.

NOHA is a stable intermediary of NO production by NOS and displays a weak competitive inhibition via interacting with the bimanganese cluster of the arginase. NOHA shows an inhibitory effect on human ARG1 with K_d_ (pH 8.5) of 3.6 μM [140] and ARG2 with K_i_ (pH 7.5) of 1.6 μM [141]. Inhibition of NOHA on ARG2 is 20 times more powerful than on ARG1 [141]. NOHA can restore NO-mediated coronary arteriolar dilation through decreasing inflammatory cells migration in IR mice [48]. Nor-NOHA, the derivative of NOHA, is also a quick-acting, potent, non-specific arginase inhibitor [142], shows an IC50 value on human ARG1 in 1.36 µM and human ARG2 in 1.26 µM [143]. Inhibition of nor-NOHA on ARG2 is 10 times stronger than on ARG1 [141]. Nor-NOHA appears to be one of the most potent inhibitors of ARG2 at physiologic pH [141]. The clinical trials of arginase inhibitors in CVDs treatment are under investigation with the use of nor-NOHA that has entered the phase 2 trials. Administration of Nor-NOHA protects from endothelial dysfunction following IR in patients with CAD (NCT02009527) [125] and ameliorates endothelium-dependent vasodilatation in patients with familial hypercholesterolemia [144] and T2DM (NCT02687152) [145]. Furthermore, nor-NOHA can rescue microcirculation of patients with HF [118] and temper CR in uremic mice [83]. Additionally, nor-NOHA is capable of attenuating the development of IR [146], hypertension [147], and atherosclerosis [148]. In particular, no apparent toxicity was present following 20 min [125] of intra-arterial infusion of nor-NOHA at a dose of 0.1 mg/min in CAD patients. And the oral bioavailability (F) of NOHA and nor-NOHA is sufficient, with F values of more than 50% [149]. Because of the hydroxyguanidine chemical and metabolic lability [149], nor-NOHA presents rapid elimination with t_1/2_ of 30 min and can be rapidly cleared from the plasma in concordance [150].

ABH and BEC, two boronic amino acid derivatives, can bind to the active manganese site of arginase. ABH has proven to be a competitive slow binding inhibitor with submicromolar activity (IC50 of 1.54 µM on human ARG1 and 2.55 µM on human ARG2) [143]. BEC is a cysteine-based ABH analogous (*K*_d_ = 270 nM for human ARG1, and *K*_i_ = 30 nM for human ARG2) [151–153]. The type and value of the inhibitions caused by boronic acid ABH and BEC depend on pH levels [154]. Emerging evidence has shown that ABH can restore the increase in BP in rats exposed to CIH [155] and decrease vascular stiffness in ApoE^−/−^ mice. ABH has high oral bioavailability (*F* = 89%), considering that almost all of the α,α-disubstituted ABH analogs presented fast clearance and weak oral bioavailability [156]. Oral administration of 400 mg ABH daily for 25 days in Fischer 344 rats does not present side effects or toxicity [157], but boronic derivatives were reported to present unacceptable toxicity against normal cells, particularly human microvascular ECs [157]. Thus, further research and more clinical studies are necessary to ensure the safety of ABH and BEC and also for ascertaining the optimum doses for prevention and treatment.

### Natural arginase inhibitors

In addition to these chemical products, amino acids and compounds derived from plants, such as polyphenols, also display an arginase inhibitory effect (Table 2).

A variety of amino acids have been proved to be weak inhibitors for arginase with IC50 values in the millimolar range [158]. Of that, L-ornithine presents the highest inhibitory efficiency (with 60% of inhibition at 20 mM and *K*_i_ of 1.02 mM on bovine arginase) by competing with substrate L-arginine on the active sites, and L-citrulline is working as an allosteric inhibitor of arginase with 53% of inhibition at 20 mM [159, 160], while norvaline is a non-competitive inhibitor of the arginase enzyme. Currently, clinical trials are underway to evaluate L-citrulline as a single candidate for the treatment of vascular endothelial dysfunction. One clinical study reported that taking L-citrulline supplements completely prevented the increase of arginase activity, and restored NO production levels in T2DM patients (NCT03358264) [161]. L-citrulline and L-ornithine also present protective effects in ischemic brain damage [162]. Intraperitoneal injection with L-norvaline for 7 days in rats with hypertension presents higher renal L-arginine level but has no significant effect on the blood concentration of amino acids [163]. Furthermore, amino acids are capable of keeping on minimum side effects that could arise from the use of chemical compounds [164].

Polyphenols, including resveratrol (RSV), piceatannol (PIC), and (-)-Epicatechin (EPI) perform ARG1 and ARG2 inhibitory activity, which have been investigated for CVDs treatment. RSV, shows arginase inhibitory activity, with inhibition of 38.7 ± 3.1% on liver ARG1 and 32.0 ± 1.3% on kidney ARG2 at 50 µmol/L [165]. RSV treatment can augment vessel constriction in de-endothelialized aortas [165], prevent hypoxia-induced human pulmonary artery SMC proliferation, and normalize right ventricular hypertrophy in an in vivo neonatal rat model of pulmonary hypertension [166]. It has also been reported to exert cardioprotective and vasoprotective effects including anti-atherosclerotic and vasorelaxation action [167]. However, high doses of RSV may cause cell death. Enhanced apoptosis was displayed in rat PASMC after intermediate concentrations of RSV (100 μM) [168]. In addition, only trace amounts have been observed following administration of an oral dose of 25 mg of RSV, with a peak value of 2 μmol/L in plasma and *t*_1/2_ of 9.2 h [169]. The oral absorption of RSV in humans is ~75%, but the bioavailability of RSV is relatively low, and oral bioavailability is less than 1% because of metabolism in the intestine and liver and its subsequent elimination [170]. PIC, an analog and metabolite of RSV [171], presents potential arginase inhibition with an inhibitory effect with IC50 of 12.1 μM on mammalian arginase [172]. PIC administration reduced the BP and recovered endothelial dysfunction in aged mice [173]. Whereas, the clinical use of PIC is restricted due to small solubility and poor bioavailability rather than medium absorption [174]. EPI has been reported to work as an uncompetitive arginase inhibitor with moderate inhibitory effect (IC50 = 1.8 ± 0.5 µM) [175]. EPI is capable of reversing EC senescence [65] and exhibiting cardioprotective effects through maintaining eNOS activity in rat models of IR [176]. Although these polyphenols only display a moderate inhibitory effect on arginase and lack specificity, those plant-derived polyphenol compounds owning to the dihydroxyphenyl group still exhibit antioxidant activity and potential cardiovascular-protective function [177].

## Conclusions and future perspective

The overactivation of arginase is the key element in the initiation and development of various CVDs. Elevated arginase expression can impair several cellular functions through regulating L-arginine metabolism and various signaling pathways, such as p38MAPK, mTOR, and p53, contributing to vascular and cardiac pathological conditions and subsequent CVDs, indicating that targeting arginase may be a potential therapeutic approach for CVDs. Recently, an increasing number of arginase inhibitors have been developed for the prevention and treatment of CVDs. Whereas, there are still unresolved questions and challenges regarding translating preclinical findings into therapeutic applications. To begin with, despite extensive clinical and experimental research indicating the potential of arginase as a biomarker and diagnostic tool for the progression of CVDs, there is still no clinical definition standard for arginase activity or ARG1/2 expression levels in blood or tissues for CVDs diagnosis. Thus, an arginase prediction model or system based on deep learning of clinical data, including CVDs types, arginase activity/expression levels, patient information, and so on, is needed to assess CVD progression, considering that ARG1 in liver is responsible for urea-cycle and ARG2 in kidney is correlated with the urine-concentrating capability, the main risk of arginase inhibitor acting systemically might induce renal dysfunction and urea-cycle disturbance, which may cause hyperammonemia. Therefore, biological monitoring of plasma ammonia and urine orotic acid, as well as hepatic function biomarkers, would be essential. Furthermore, the global loss of ARG2 activity may contribute to the development of hypertension [178]. Thus, exploring ARG1- and ARG2-specific inhibitors must be investigated. However, no isoform-specific arginase inhibitor has been reported owing to the substantial similarity between ARG1 and ARG2. Inspiringly, emerging evidence has shown the crucial role of vaccines in cardiovascular disease, which can prevent CVDs onset and development through immunomodulation [179], and vaccines against ARG1 or ARG2 is already in preliminary trials for cancer treatment [180]. Whereas, neither ARG1 nor ARG2-targeting vaccines were reported in CVDs therapy until now. It is worth noting that ARG2 regulates vascular cell functions through mTOR, p66shc, and p53 signaling pathways independently of its L-arginine-urea hydrolase activity, implying that inhibiting the level of arginase expression may be a more effective approach for CVDs therapy. In addition, a number of arginase inhibitors, especially those containing boronic acid functionality may cause acute toxicity symptoms in human, such as nausea, vomiting, diarrhea, and lethargy [157]. Therefore, the effect of arginase inhibitors in larger clinical settings and the potential adverse effects of long-term arginase inhibitor treatment needs to be determined. Of note, some compounds isolated from natural medicinal plants can act as arginase inhibitors with hypotoxicity, which may provide a novel approach for arginase inhibition.

## Supplementary information

Responses to Initial Quality Check

Combined confirmation e-mail
