# Supplementary material for: Arginase: shedding light on the mechanisms and opportunities in cardiovascular diseases
Source: Cell Death Discov. 2022 Oct 8;8:413. doi: 10.1038/s41420-022-01200-4 (PMC9547100; doi:10.1038/s41420-022-01200-4)
Supplement: Supplementary file 1 — Responses to Initial Quality Check [file 41420_2022_1200_MOESM1_ESM.docx]

**Responses to Initial Quality Check**

1. Please make sure all corresponding authors link their own ORCID.

We apologized for this omission. All corresponding authors have linked their own ORCID to their account on MTS following the instructions in the link. <https://www.springernature.com/gp/researchers/orcid/orcid-for-nature-research>

2. Please make sure all figures are cited within the article file: Fig 1 has not been cited.

This is a misunderstanding. Fig 1 has been cited in the article file of the last submission. (please see the page 6, line 127)

3. It has come to our attention that your most recent author list differs from the one in your original submission. Please request agreement from all authors including additions and deletions, these can be collected in the following way:

Email your co-authors with the change, and ask them to reply to your email confirming that they agree to these changes. Once you have collected these replies, please combine all of the co-authors’ email responses in one document and upload this file to your submission.

We have emailed our co-authors for the confirmation of these changes. All authors have replied and agreed to these changes. All of the co-authors’ email responses were collected in a PDF named “Combined confirmation e-mail”, and uploaded as a supplementary material in our submission.
