# Supplementary material for: Arginase: shedding light on the mechanisms and opportunities in cardiovascular diseases
Source: Cell Death Discov. 2022 Oct 8;8:413. doi: 10.1038/s41420-022-01200-4 (PMC9547100; doi:10.1038/s41420-022-01200-4)
Supplement: Supplementary file 2 — Combined confirmation e-mail [file 41420_2022_1200_MOESM2_ESM.pdf]

From: 李卓卓 lizhuozhuo2021@163.com
Subject: Re:Authors list changes confirmation request
Date: September 22, 2022 at 9:41 AM
To: Yuyan Xiong yuyan.xiong@nwu.edu.cn

李

Dear Prof. Dr. Yuyan Xiong,

I agree with these changes of the authors list.

Sincerely,

Zhuozhuo Li

At 2022-09-21 22:36:10, "Yuyan Xiong" <yuyan.xiong@nwu.edu.cn> wrote:

> Dear Co-authors of Manuscript CDDISCOVERY-22-4860R1,
>
> Since Miss.Yaoyao Huang, Miss.Wenxuan Liu and Mr. Ziwei Lv were involved in the revision work of our submitted manuscript CDDISCOVERY-22-4860R1 entitled "Arginase: shedding light on the mechanisms and opportunities in cardiovascular diseases", we need to add these three authors Yaoyao Huang, Wenxuan Liu and Ziwei Lv into the co-authors list of our submitted manuscript according to the author contribution role. Currently, the authors list is Zhuozhuo Li, Liwei Wang, Yuanyuan Ren, Yaoyao Huang, Wenxuan Liu, Ziwei Lv, Lu Qian, Yi Yu, and Yuyan Xiong.
>
> As the corresponding author, I am writing this e-mail to confirm whether you agree with these changes of the authors list?
>
> Looking forward to your early reply.
>
> Kind regards,
>
> Yuyan Xiong
>
> Prof. Dr. Yuyan Xiong,
> Faculty of Life and Science and Medicine
> Northwest University
> Xi'an 70069, Shaanxi, China
> yuyan.xiong@nwu.edu.cn
> TEL: 0086-15691961676

From: 西北大学医学院临床医学王利玮 q13031877623@163.com
Subject: Re: Authors list changes confirmation request
Date: September 22, 2022 at 11:37 AM
To: yuyan.xiong yuyan.xiong@nwu.edu.cn

Dear Professor Xiong,

Yes，I agree with these changes.

With kind regards,

Liwei Wang

---- Replied Message ----

From Yuyan Xiong<yuyan.xiong@nwu.edu.cn>

Date 09/21/2022 22:36

To lizhuozhuo2021@163.com<lizhuozhuo2021@163.com>、q13031877623@163.com<q13031877623@163.com>、Ren Yuanyuan<renyuanyuan1221@163.com>、1072554547@qq.com<1072554547@qq.com>、1837543199@qq.com<1837543199@qq.com>、635955670@qq.com<635955670@qq.com>、2640933799@qq.com<2640933799@qq.com>、yiyu@nwu.edu.cn<yiyu@nwu.edu.cn>

Subject Authors list changes confirmation request

Dear Co-authors of Manuscript CDDISCOVERY-22-4860R1,
Since Miss.Yaoyao Huang, Miss.Wenxuan Liu and Mr. Ziwei Lv were involved in the revision work of our submitted manuscript CDDISCOVERY-22-4860R1 entitled "Arginase: shedding light on the mechanisms and opportunities in cardiovascular diseases", we need to add these three authors Yaoyao Huang, Wenxuan Liu and Ziwei Lv into the co-authors list of our submitted manuscript according to the author contribution role. Currently, the authors list is Zhuozhuo Li, Liwei Wang, Yuanyuan Ren, Yaoyao Huang, Wenxuan Liu, Ziwei Lv, Lu Qian, Yi Yu, and Yuyan Xiong.

As the corresponding author, I am writing this e-mail to confirm whether you agree with these changes of the authors list?

Looking forward to your early reply.

Kind regards,

Yuyan Xiong

Prof. Dr. Yuyan Xiong,
Faculty of Life and Science and Medicine
Northwest University
Xi'an 70069, Shaanxi, China
yuyan.xiong@nwu.edu.cn
TEL: 0086-15691961676

From: 任媛媛 renyuanyuan1221@163.com
Subject: Re:Authors list changes confirmation request
Date: September 22, 2022 at 9:42 AM
To: Yuyan Xiong yuyan.xiong@nwu.edu.cn

R

Dear Prof. Dr. Yuyan Xiong,

Yes，I agree with these changes.

Sincerely yours,

Yuanyuan Ren

At 2022-09-21 22:36:10, "Yuyan Xiong" <yuyan.xiong@nwu.edu.cn> wrote:

> Dear Co-authors of Manuscript CDDISCOVERY-22-4860R1,
>
> Since Miss.Yaoyao Huang, Miss.Wenxuan Liu and Mr. Ziwei Lv were involved in the revision work of our submitted manuscript CDDISCOVERY-22-4860R1 entitled "Arginase: shedding light on the mechanisms and opportunities in cardiovascular diseases", we need to add these three authors Yaoyao Huang, Wenxuan Liu and Ziwei Lv into the co-authors list of our submitted manuscript according to the author contribution role. Currently, the authors list is Zhuozhuo Li, Liwei Wang, Yuanyuan Ren, Yaoyao Huang, Wenxuan Liu, Ziwei Lv, Lu Qian, Yi Yu, and Yuyan Xiong.
>
> As the corresponding author, I am writing this e-mail to confirm whether you agree with these changes of the authors list?
>
> Looking forward to your early reply.
>
> Kind regards,
>
> Yuyan Xiong
>
> Prof. Dr. Yuyan Xiong,
> Faculty of Life and Science and Medicine

[illegible]
Northwest University
Xi'an 70069, Shaanxi, China
yuyan.xiong@nwu.edu.cn
TEL: 0086-15691961676

From: **huangyaoyao** 1072554547@qq.com
Subject: Re: Authors list changes confirmation request
Date: September 21, 2022 at 11:53 PM
To: Yuyan Xiong yuyan.xiong@nwu.edu.cn

H

Dear Prof. Dr. Xiong,

Yes，I agree with these changes.

Yaoyao Huang

---Original---

From: "Yuyan Xiong"<yuyan.xiong@nwu.edu.cn>
Date: Wed, Sep 21, 2022 22:36 PM
To: "lizhuozhuo2021"<lizhuozhuo2021@163.com>;"q13031877623"<q13031877623@163.com>;"Ren Yuanyuan"<renyuanyuan1221@163.com>;"1072554547"<1072554547@qq.com>;"1837543199"<1837543199@qq.com>;"635955670"<635955670@qq.com>;"2640933799"<2640933799@qq.com>;"yiyu"<yiyu@nwu.edu.cn>;
Subject: Authors list changes confirmation request

Dear Co-authors of Manuscript CDDISCOVERY-22-4860R1,
Since Miss.Yaoyao Huang, Miss.Wenxuan Liu and Mr. Ziwei Lv were involved in the revision work of our submitted manuscript CDDISCOVERY-22-4860R1 entitled "Arginase: shedding light on the mechanisms and opportunities in cardiovascular diseases", we need to add these three authors Yaoyao Huang, Wenxuan Liu and Ziwei Lv into the co-authors list of our submitted manuscript according to the author contribution role. Currently, the authors list is Zhuozhuo Li, Liwei Wang, Yuanyuan Ren, Yaoyao Huang, Wenxuan Liu, Ziwei Lv, Lu Qian, Yi Yu, and Yuyan Xiong.

As the corresponding author, I am writing this e-mail to confirm whether you agree with these changes of the authors list?

Looking forward to your early reply.

Kind regards,

Yuyan Xiong

Prof. Dr. Yuyan Xiong,
Faculty of Life and Science and Medicine
Northwest University
Xi'an 70069, Shaanxi, China
yuyan.xiong@nwu.edu.cn
TEL: 0086-15691961676

From: ??? 1837543199@qq.com
Subject: Re:Authors list changes confirmation request
Date: September 22, 2022 at 4:24 PM
To: Yuyan Xiong yuyan.xiong@nwu.edu.cn

W

Dear Prof. Dr. Yuyan Xiong,

I agree with these changes of the authors list.

Sincerely,

Wenxuan Liu

------------------ Original ------------------

From: "Yuyan Xiong" <yuyan.xiong@nwu.edu.cn>;
Date: Wed, Sep 21, 2022 10:36 PM
To: "lizhuozhuo2021"<lizhuozhuo2021@163.com>;"q13031877623"<q13031877623@163.com>;"Ren Yuanyuan"<renyuanyuan1221@163.com>;"1072554547"<1072554547@qq.com>;"???" <1837543199@qq.com>;"635955670"<635955670@qq.com>;"2640933799" <2640933799@qq.com>;"yiyu"<yiyu@nwu.edu.cn>;
Subject: Authors list changes confirmation request

Dear Co-authors of Manuscript CDDISCOVERY-22-4860R1,
Since Miss.Yaoyao Huang, Miss.Wenxuan Liu and Mr. Ziwei Lv were involved in the revision work of our submitted manuscript CDDISCOVERY-22-4860R1 entitled "Arginase: shedding light on the mechanisms and opportunities in cardiovascular diseases", we need to add these three authors Yaoyao Huang, Wenxuan Liu and Ziwei Lv into the co-authors list of our submitted manuscript according to the author contribution role. Currently, the authors list is Zhuozhuo Li, Liwei Wang, Yuanyuan Ren, Yaoyao Huang, Wenxuan Liu, Ziwei Lv, Lu Qian, Yi Yu, and Yuyan Xiong.

As the corresponding author, I am writing this e-mail to confirm whether you agree with these changes of the authors list?

Looking forward to your early reply.

Kind regards,

Yuyan Xiong

Prof. Dr. Yuyan Xiong,
Faculty of Life and Science and Medicine
Northwest University
Xi'an 70069, Shaanxi, China
yuyan.xiong@nwu.edu.cn
TEL: 0086-15691961676

From: ♤♧♡◇ 635955670@qq.com
Subject: Re:Authors list changes confirmation request
Date: September 22, 2022 at 4:25 PM
To: Yuyan Xiong yuyan.xiong@nwu.edu.cn

Dear Prof. Dr. Yuyan Xiong,

Yes, I agree with these changes.

Sincerely,

Ziwei Lv

------------------ Original ------------------

From: "Yuyan Xiong" <yuyan.xiong@nwu.edu.cn>;
Date: Wed, Sep 21, 2022 10:36 PM
To: "lizhuozhuo2021"<lizhuozhuo2021@163.com>;"q13031877623"<q13031877623@163.com>;"Ren Yuanyuan"<renyuanyuan1221@163.com>;"1072554547"<1072554547@qq.com>;"1837543199"<1837543199@qq.com>;"♤♧♡◇"<635955670@qq.com>;"2640933799"<2640933799@qq.com>;"yiyu"<yiyu@nwu.edu.cn>;
Subject: Authors list changes confirmation request

Dear Co-authors of Manuscript CDDISCOVERY-22-4860R1,
Since Miss.Yaoyao Huang, Miss.Wenxuan Liu and Mr. Ziwei Lv were involved in the revision work of our submitted manuscript CDDISCOVERY-22-4860R1 entitled "Arginase: shedding light on the mechanisms and opportunities in cardiovascular diseases", we need to add these three authors Yaoyao Huang, Wenxuan Liu and Ziwei Lv into the co-authors list of our submitted manuscript according to the author contribution role. Currently, the authors list is Zhuozhuo Li, Liwei Wang, Yuanyuan Ren, Yaoyao Huang, Wenxuan Liu, Ziwei Lv, Lu Qian, Yi Yu, and Yuyan Xiong.

As the corresponding author, I am writing this e-mail to confirm whether you agree with these changes of the authors list?

Looking forward to your early reply.

Kind regards,

Yuyan Xiong

Prof. Dr. Yuyan Xiong,
Faculty of Life and Science and Medicine
Northwest University
Xi'an 70069, Shaanxi, China
yuyan.xiong@nwu.edu.cn
TEL: 0086-15691961676

From: 钱露 2640933799@qq.com
Subject: Re:Authors list changes confirmation request
Date: September 22, 2022 at 9:31 AM
To: Yuyan Xiong yuyan.xiong@nwu.edu.cn

Dear Prof. Yuyan Xiong,

I agree with these changes of the authors list.

Kind regards,
Lu Qian

------------------ Original ------------------

From: "Yuyan Xiong" <yuyan.xiong@nwu.edu.cn>;
Date: Wed, Sep 21, 2022 10:36 PM
To: "lizhuozhuo2021"<lizhuozhuo2021@163.com>;"q13031877623"<q13031877623@163.com>;"Ren Yuanyuan"<renyuanyuan1221@163.com>;"1072554547"<1072554547@qq.com>;"1837543199" <1837543199@qq.com>;"635955670"<635955670@qq.com>;"钱露"<2640933799@qq.com>;"yiyu" <yiyu@nwu.edu.cn>;
Subject: Authors list changes confirmation request

Dear Co-authors of Manuscript CDDISCOVERY-22-4860R1,
Since Miss.Yaoyao Huang, Miss.Wenxuan Liu and Mr. Ziwei Lv were involved in the revision work of our submitted manuscript CDDISCOVERY-22-4860R1 entitled "Arginase: shedding light on the mechanisms and opportunities in cardiovascular diseases", we need to add these three authors Yaoyao Huang, Wenxuan Liu and Ziwei Lv into the co-authors list of our submitted manuscript according to the author contribution role. Currently, the authors list is Zhuozhuo Li, Liwei Wang, Yuanyuan Ren, Yaoyao Huang, Wenxuan Liu, Ziwei Lv, Lu Qian, Yi Yu, and Yuyan Xiong.

As the corresponding author, I am writing this e-mail to confirm whether you agree with these changes of the authors list?

Looking forward to your early reply.

Kind regards,

Yuyan Xiong

Prof. Dr. Yuyan Xiong,
Faculty of Life and Science and Medicine
Northwest University
Xi'an 70069, Shaanxi, China
yuyan.xiong@nwu.edu.cn
TEL: 0086-15691961676

From: 于怡 yiyu@nwu.edu.cn
Subject: Re: Authors list changes confirmation request
Date: September 21, 2022 at 10:57 PM
To: Yuyan Xiong yuyan.xiong@nwu.edu.cn

于

Dear Prof. Xiong,
I am aware of the changes of the authors list, and agree to add Yaoyao Huang, Wenxuan Liu and Ziwei Lv as co-authors in manuscript CDDISCOVERY-22-4860R1 entitled "Arginase: shedding light on the mechanisms and opportunities in cardiovascular diseases".

Sincerely yours,
Dr. Yi Yu
Faculty of Life and Science and Medicine
Northwest University

-----原始邮件-----
发件人:"Yuyan Xiong" <yuyan.xiong@nwu.edu.cn>
发送时间:2022-09-21 22:36:10 (星期三)
收件人: lizhuozhuo2021@163.com, q13031877623@163.com, "Ren Yuanyuan" <renyuanyuan1221@163.com>, 1072554547@qq.com, 1837543199@qq.com, 635955670@qq.com, 2640933799@qq.com, yiyu@nwu.edu.cn
抄送:
主题: Authors list changes confirmation request

Dear Co-authors of Manuscript CDDISCOVERY-22-4860R1,
Since Miss.Yaoyao Huang, Miss.Wenxuan Liu and Mr. Ziwei Lv were involved in the revision work of our submitted manuscript CDDISCOVERY-22-4860R1 entitled "Arginase: shedding light on the mechanisms and opportunities in cardiovascular diseases", we need to add these three authors Yaoyao Huang, Wenxuan Liu and Ziwei Lv into the co-authors list of our submitted manuscript according to the author contribution role. Currently, the authors list is Zhuozhuo Li, Liwei Wang, Yuanyuan Ren, Yaoyao Huang, Wenxuan Liu, Ziwei Lv, Lu Qian, Yi Yu, and Yuyan Xiong.

As the corresponding author, I am writing this e-mail to confirm whether you agree with these changes of the authors list?

Looking forward to your early reply.

Kind regards,

Yuyan Xiong

Prof. Dr. Yuyan Xiong,
Faculty of Life and Science and Medicine
Northwest University
Xi'an 70069, Shaanxi, China
yuyan.xiong@nwu.edu.cn
TEL: 0086-15691961676
